# The protocol for the prehabilitation for thoracic surgery study: a randomized pragmatic trial comparing a short home-based multimodal program to aerobic training in patients undergoing video-assisted thoracoscopic surgery lobectomy

**DOI:** 10.1186/s13063-023-07220-4

**Published:** 2023-03-15

**Authors:** Yuchao Liu, Zijia Liu, Yuelun Zhang, Yushang Cui, Lijian Pei, Yuguang Huang

**Affiliations:** 1 Department of Anesthesiology, Chinese Academy of Medical Sciences & Peking Union Medical College Hospital, Beijing, 100730 China; 2 Department of Medical Research Center, Chinese Academy of Medical Sciences & Peking Union Medical College Hospital, Beijing, 100730 China; 3 Department of Thoracic Surgery, Chinese Academy of Medical Sciences & Peking Union Medical College Hospital, Beijing, 100730 China

**Keywords:** Multimodal prehabilitation, Aerobic training, Video-assisted thoracoscopic surgeries, Lobectomy, Pragmatic trial, Functional capacity, 6-min walk distance

## Abstract

**Background:**

Prehabilitation has been shown to have a positive effect on the postoperative recovery of functional capacity in patients undergoing video-assisted thoracoscopic surgery (VATS) lobectomy. The optimal way to implement prehabilitation programs, such as the optimal forms of prehabilitation, duration, intensity, and methods to improve compliance, remained to be studied. This Prehabilitation for Thoracic Surgery Study will compare the effectiveness of multimodal and aerobic training-only programs in patients undergoing thoracoscopic lobectomy.

**Methods:**

This randomized pragmatic trial will be conducted in Peking Union Medical College Hospital (PUMCH) and include 100 patients who are eligible to undergo VATS lobectomy. Patients will be randomized to a multimodal or aerobic training group. Prehabilitation training guidance will be provided by a multidisciplinary care team. The patients in the multimodal group will perform aerobic exercises, resistance exercises, breathing exercises, psychological improvement strategies, and nutritional supplementation. Meanwhile, the patients in the aerobic group will conduct only aerobic exercises. The interventions will be home-based and supervised by medical providers. The patients will be followed up until 30 days after surgery to investigate whether the multimodal prehabilitation program differs from the aerobic training program in terms of the magnitude of improvement in functional capability pre- to postoperatively. The primary outcome will be the perioperative 6-min walk distance (6MWD). The secondary outcomes will include the postoperative pulmonary functional recovery status, health-related quality of life score, incidence of postoperative complications, and clinical outcomes.

**Discussion:**

Prehabilitation remains a relatively new approach that is not widely performed by thoracic surgery patients. The existing studies mainly focus on unimodal interventions. While multimodal prehabilitation strategies have been shown to be preferable to unimodal strategies in a few studies, the evidence remains scarce for thoracic surgery patients. The results of this study will contribute to the understanding of methods for thoracoscopic lobectomy patients.

**Trial registration:**

ClinicalTrials.gov NCT04049942. Registered on August 8, 2019.

**Supplementary Information:**

The online version contains supplementary material available at 10.1186/s13063-023-07220-4.

## Background

Lung cancer remains the leading cause of cancer-related deaths worldwide [1]. For localized non-small cell lung cancer (NSCLC), surgical resection with radical lymph node dissection is a potentially curative option [2]. Surgical techniques have advanced, and video-assisted thoracoscopic surgery (VATS) is currently a well-established and preferred choice at many institutions due to its minimally invasive nature [3]. However, the incidence of postoperative pulmonary complications (PPCs) is still reported to be 12–40% [4, 5].

The concept of prehabilitation, the process of optimizing the functional capacity of an individual before surgery, has been developed to improve the outcomes of surgical patients [6]. Although various unimodal and multimodal prehabilitation strategies have been shown to improve functional capacity in patients undergoing gastrointestinal surgeries [6–11], pursuing an optimal strategy remains a work in progress, especially in the thoracic surgery community.

Until recently, most of the preoperative interventions that have been implemented in thoracic surgery patients have been unimodal, and the majority of previous studies have focused on exercise-based training programs, particularly aerobic exercises [12–14]. Although there is increasing evidence that preoperative exercise training improves physical function and pulmonary function, it remains unclear how exercise training compares to other forms of prehabilitation in efficacy and whether improvements in functional capacity lead to better postoperative outcomes.

Our team conducted a randomized controlled trial in 2017 (registration number NCT03068507) to examine the efficacy of a 2-week multimodal home-based prehabilitation strategy in patients undergoing thoracoscopic surgeries for lung cancer. The multimodal program included physical exercise, respiratory training, nutritional supplements, and psychological optimization. This study yielded promising results, showing that a short-term home-based multimodal prehabilitation program could improve perioperative functional capacity in patients undergoing thoracoscopic lobectomy, as indicated by an improved perioperative 6-min walk distance (6MWD) [15]. The average 6MWD was 60.9 m higher perioperatively in the prehabilitation group compared to the control group (95% confidence interval [CI], 32.4–89.5; *P* < 0.001).

The Prehabilitation for Thoracic Surgery Study aims to further understand the role of prehabilitation in thoracoscopic surgery patients by comparing the efficacy of a short multimodal home-based prehabilitation program with an aerobic exercise-only prehabilitation program, as the latter has been most extensively investigated to date.

### Research question

The aim of the Prehabilitation for Thoracic Surgery Study is to answer the following question: Is short-term multimodal prehabilitation more beneficial than aerobic exercise-based prehabilitation in improving perioperative functional walking capacity in patients undergoing VATS lobectomy?

## Methods

### Trial design

**Fig. 1:**
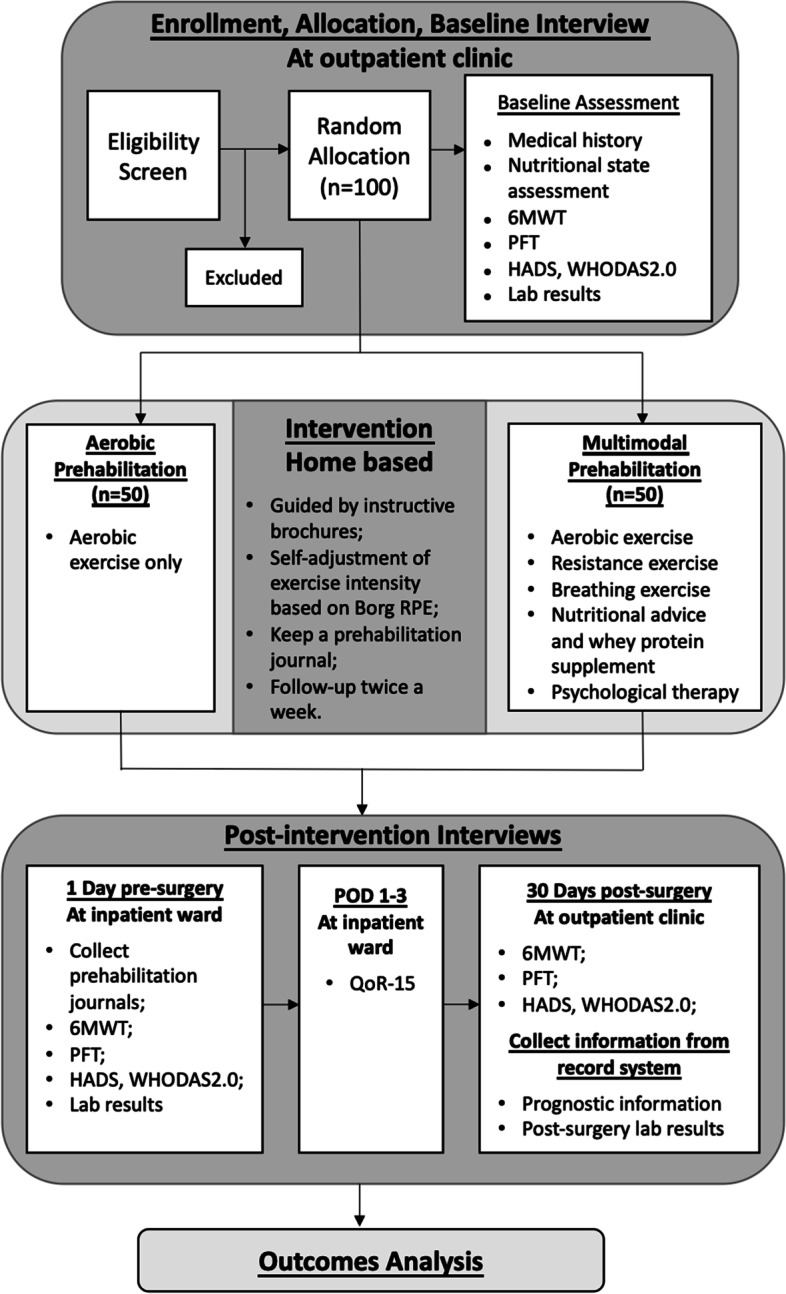
Flow diagram of the study. 6MWT, 6-min walk test; PFT, pulmonary function test; HADS, Hospital Anxiety and Depression Scale; WHODAS 2.0, World Health Organization Disability Assessment Schedule II; Borg RPE, Borg Rating of Perceived Exertion Scale; POD, postoperative day; QoR-15, Quality of Recovery Score-15

The Prehabilitation for Thoracic Surgery Study is a randomized pragmatic trial comparing the impact of a short home-based multimodal prehabilitation program to a preoperative aerobic training program in patients undergoing VATS for lung cancer. The overall trial design is illustrated in Fig. 1 (see Additional file 1 for the Standard Protocol Items: Recommendations for Interventional Trials (SPIRIT) Checklist). Ethics board approval of this study was obtained from the Peking Union Medical College Hospital (PUMCH) Institutional Review Board on July 23, 2019. This study will be conducted in PUMCH by a multidisciplinary care team, including anesthesiologists, thoracic surgeons, nutritionists, and rehabilitation medicine practitioners. After they are recruited and provided consent, the patients will be randomized to two study arms; then, they will be instructed to undergo either a tailored aerobic program or a multimodal prehabilitation program during the period of time prior to surgery. The patients will have a 30-day follow-up after surgery. The effects of the two arms will be assessed on the basis of physical capacity test results (6MWD), pulmonary function test results, patient-reported qualitative outcomes, clinical outcomes, and perioperative laboratory results. The patients, care providers, and researchers in charge of enrolling, following up, and assessing outcomes (performing the 6MWT) will not be blinded to the allocation due to the nature of the intervention. Data analysts will be blinded. Generally, data analysts will not need to be unblinded.

### Participant eligibility criteria

The participating patients must be (1) thoracic surgery outpatients in PUMCH, (2) aged from 18 to 70 years, (3) suspected of having lung cancer, and (4) scheduled for elective thoracoscopic surgery in PUMCH. Patients will be excluded from the study if they (1) decline to participate in the study (due to any reason), (2) have an American Society of Anesthesiologists (ASA) grade > III, (3) plan to undergo or have undergone neoadjuvant therapy, (4) are unable to tolerate any component of the multimodal prehabilitation or aerobic program (including exercises, whey protein supplements, and functional capacity tests), or (5) have other severe cardiopulmonary diseases that may affect the 6MWD results.

### Patient recruitment and allocation

In PUMCH, approximately 100–150 VATS lobectomy surgeries are scheduled each month. Patients will be recruited from the thoracic surgery outpatient department where they receive consults before surgery. When they are scheduled for VATS lobectomy, they will be screened for eligibility and asked to provide informed consent by a research staff. The patients included will then be randomly assigned to two equally sized groups, the multimodal prehabilitation group and the aerobic prehabilitation group, on the basis of a computer-generated sequence. The random allocation results will be concealed using sealed opaque envelopes. One of the main researchers of the study designs the statistical analysis plans and will be generating the allocation sequence. Two experienced anesthesiologist researchers will be in charge of enrolling and assigning participants according to the randomization result.

### Baseline interview

Upon patient recruitment and allocation to groups, a one-on-one baseline interview and evaluation will be conducted to (1) record patients’ comprehensive medical history; (2) assess patients’ nutritional states; (3) assess patients’ functional capacity during the 6-min walk test (6MWT); (4) conduct a pulmonary function test (Spirodoc portable pulmonary function meter, Medical International Research S.r.l., 09,000,261,972), during which the forced expiratory volume in 1 s (FEV1, L), FEV1%, forced vital capacity (FVC, L), FVC%, FEV1/FVC, peak expiratory flow (PEF, L/s), and PEF% will be recorded; (5) record laboratory results, if applicable, including the levels of alanine transaminase (ALT), creatine (CR), C-reactive protein (CRP), albumin (Alb), glucose (Glu), and myocardial enzymes; (6) evaluate the patients’ baseline quality of life and mental state using standard tools [hospital anxiety and depression scale (HADS), World Health Organization disability assessment schedule II (WHODAS 2.0) score, postoperative quality of recovery score-15 (QoR-15)]; (7) explain in detail the prehabilitation plan for both groups; and (8) encourage patients to choose a preferred way to be followed up, such as by telephone, short text messages, or WeChat messaging.

### Interventions

Aside from the prehabilitation strategies, all patients enrolled will receive conventional perioperative recommendations, including those based on preoperative anesthesia assessments, drug treatment recommendations for chronic diseases, and recommendations to quit smoking and practice abstinence. The patients will also be provided information on anesthesia and surgical processes, according to each individual’s needs.

We will encourage the patients in both groups to choose their preferred types of exercise and form individual-specific prehabilitation plans. A prehabilitation instruction brochure will be distributed to patients along with a daily self-evaluation prehabilitation journal, where they can record their adherence to the prehabilitation plans. Standardized short message interviews will be sent to patients twice a week in the manner chosen by the patients to optimize adherence and promote timely feedback. The patients will receive intraoperative and perioperative care according to standard practice in terms of surgical procedures, anesthesia and analgesia management, and nursing care.

The patients will be encouraged to reach out to our researchers if there are injuries or discomforts during the follow-up period. Since the intervention is based on moderate exercise and nutrition supplements, and exercise plan is individualized to consider participants’ capacity and preference, harm of the intervention is considered minimal. However, if patients experience injuries or discomforts due to the exercises or whey protein supplement, the intervention will be discontinued and we plan to offer appropriate medical and financial support. At any point in the study period, if patients request to stop or pause the intervention, the intervention will be discontinued.

#### Multimodal prehabilitation strategy features

For the multimodal group, we will implement the following prehabilitation strategy:

1. A 30-min aerobic exercise session will be performed at least 3 times a week; it will be divided into a 5-min warm-up (including ankle extensor lifts and static quadriceps contraction) and a 25-min aerobic exercise in the form of jogging, power walking, or cycling, depending on the patients’ individual choice. We will encourage patients to tailor their exercise intensity to the Borg Rating of Perceived Exertion (RPE) Scale [16] shown in Table 1 and target heart rate (HR), increasing or decreasing the intensity according to the level of exertion throughout the prehabilitation period. We will recommend that patients achieve moderate to high levels of exertion, as indicated by 13–16 points on the Borg RPE Scale and a target HR of (220 – age − resting HR) × 70% + resting HR. The methods of using the Borg RPE Scale and target HR to adjust the exercise intensity will be explained at the baseline interview, and both methods will be illustrated in the brochure we give the patients. We will also provide each patient with a pulse oximeter (Jiangsu Yuyue Medical Equipment & Supply Co., Ltd., 20172201070) to help monitor HR. For patients who choose an outdoor activity as their aerobic exercise training method, we will stress the importance of safety and advise them to avoid exercising outdoors on days with poor air quality index values (> 100).
2. Guided resistance exercises will be performed at least twice a week. At the baseline interview, a research staff qualified in performing physical therapy training and evaluations will initially evaluate the physical status of the patients and then explain and demonstrate the resistance exercises. We will provide each patient in the multimodal group with two pulling straps of different elasticities. Resistance training will be performed with either strap, depending on the patient’s individual strength limits. Most patients will start training with the less elastic strap and advance to the more elastic strap once they feel that training with the less elastic strap requires too little effort. We will assign 4 pre-set resistance postures for the training sessions, involving 4 major muscle groups (upper and lower limbs, chest, and core muscles). The complete resistance training session will include 3 sets of exercises with an interval of 2 min between each set. Each set will include 10–12 repetitions of the 4 resistance postures. At the end of each complete session, the patients will be asked to evaluate their perceived exertion by using the Borg RPE Scale. The target score will be 13–16. The number of repetitions performed within each set and the strap selection should be adjusted on an individual basis throughout the prehabilitation period according to the Borg RPE scores.
3. Breathing exercises will be performed at least 3 times per day, with each session lasting more than 10 min. We will introduce three types of exercises: (1) guided effective coughing (sit up and lean slightly forward, inhale fully then cough out in a short and forceful manner while engaging the core muscles), (2) blowing up a small balloon in one breath and blowing it up slowly for more than 5 s, and (3) breathing training with the Tri-Ball Respiratory Exerciser (Leventon S.A., Barcelona, Spain) that we will distribute. All three types of breathing exercises will be demonstrated at the baseline interview and explained in the brochure. The patients will be allowed to choose to conduct one or more types of breathing exercises in one session.
4. Regarding nutritional advice and whey protein supplementation, patients’ nutritional status and dietary habits will be evaluated at baseline by a research staff member trained in nutriology. Nutritional advice predetermined by our nutritionists will be given to modulate the patient’s eating habits, mainly aiming at reducing fat-rich diets and increasing high-quality protein uptake. Patients with malnutrition will be advised to increase their calorie uptake. We will also provide patients in the multimodal group with whey protein powder (Inerish; Sino-American Medical Institute Inc., San Diego, CA) as a protein supplement and advise them to take it daily (20 g/day for males and 15 g/day for females). If patients plan on performing exercises that day, we will advise that the whey protein powder is taken within 1 h postexercise, promoting muscle synthesis.
5. Psychological therapy will include listening to soothing music, broadcasts, or other relaxing activities of the patients’ choice before sleep. We will provide each patient in the multimodal group with a simple music player with prerecorded relaxing, classical music tracks and encourage patients to use it if they prefer this type of relaxation method. We will also encourage patients to choose their preferred methods of relaxation, including listening to audiobooks, meditating, and watching relaxing television shows.

**Table 1:** Borg RPE Scale

Score	Level of exertion
6	No exertion at all
7	Extremely light
8	Minimal recognition of effort
9	Very light (equivalent to walking at your own pace for several minutes)
10	Could just start to beware of breathing
11	Fairly light (conversation is easy and feel like you could keep the pace for a long time)
12	Light
13	Somewhat hard, but still feel able to continue
14	You could hear your breathing clearly but not struggling
15	Hard (you could talk but not in full sentences)
16	Heavy
17	Very hard (you could continue but would be pushing yourself to feel very fatigued)
18	You could not talk because of the heavy panting
19	Extremely hard
20	Maximal exertion

#### Aerobic prehabilitation strategy features

The aerobic group will receive the same instructions regarding the individualized home-based aerobic exercise program as the multimodal group will, but they will not receive advice regarding the other aspects of their behaviors. These patients will also be advised to record their daily exercises in a prehabilitation journal and will receive the same follow-up interviews twice a week.

### Outcomes

The primary outcome will be the perioperative 6MWD (baseline, 1 day presurgery, and 30 days postsurgery). The 6MWT will be conducted following the guidelines of the American Thoracic Society (ATS) [17]. The research staff members who will be involved in performing the 6MWT will all be trained before administering this test. We will conduct the 6MWT indoors in a 30-m-long corridor in PUMCH. We will ensure that supplies needed for safety issues are readily available whenever the 6MWT is performed, including oxygen, sublingual nitroglycerine, aspirin, and albuterol. All the staff members who will be involved have been previously certified in cardiopulmonary resuscitation. The 6MWT will be conducted in a standard manner. From the 10-min rest period before the test to all the verbal instructions provided to the patients before and during the test, all procedures will be performed in accordance with the standard ATS guidelines.

We will record the patients’ sex, age, height, weight, medications before the test, and whether they need oxygen supplementation during the test. We will measure blood pressure, HR, oxygen saturation, and the Borg RPE score before and after the test. We will note the number of laps and calculate the 6MWD.

The secondary outcomes will include the perioperative pulmonary function test results, including the FEV1, FEV1%, FVC, FVC%, FEV1/FVC, PEF, and PEF% values (baseline, 1 day presurgery, and 30 days postsurgery); the perioperative HADS and WHODAS 2.0 (baseline, 1 day presurgery, and 30 days postsurgery); post-surgery QoR-15 (3 consecutive days after surgery); prognostic information (mortality and morbidity, length of hospital stay, ICU stay time, duration of chest tube placement duration); and perioperative laboratory results, if applicable, including the levels of ALT, CR, CRP, Alb, Glu, and myocardial enzymes (baseline, 1 day presurgery, and 30 days postsurgery).

### Implementation timeline

**Fig. 2:**
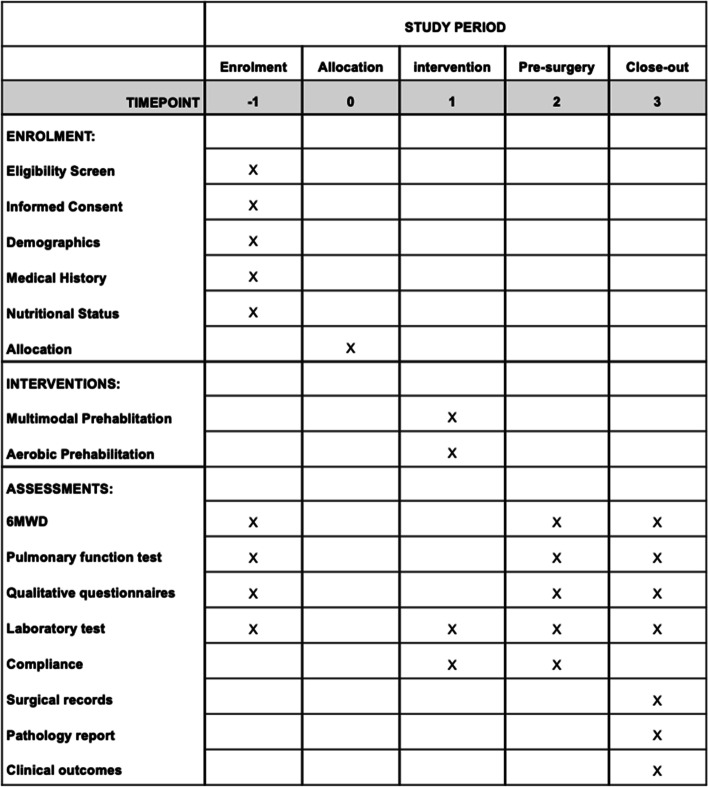
Timeline according to the SPIRIT guidelines. Time points: − 1, enrollment at thoracic outpatient clinic; 0, allocation; 1, waiting period before surgery; 2, day before surgery; and 3, 30 days after surgery. The pulmonary function test results will include the FEV1, FEV1%, FVC, FVC%, FEV1/FVC, PEF, and PEF% values; the qualitative questionnaires include the HADS, World Health Organization Disability Assessment Schedule II (WHODAS 2.0), and Postoperative Quality of Recovery-15 (QoR-15); the laboratory test results include the levels of ALT, CR, CRP, Alb, Glu, and myocardial enzymes; the clinical outcomes include the mortality and morbidity, length of hospital stay, ICU stay time, and duration of chest tube placement duration

The timeline of this study is 6 to 8 weeks; prehabilitation will be implemented for 2 to 4 weeks before surgery, and the follow-up period will include the 4 weeks after surgery. The timeline will differ across individuals, mainly due to the wait time before surgery. Clinical outcome data will be retrieved by practitioners from the inpatient records within 1 month after surgery. Figure 2 shows the full timeline.

### Sample size

In this study, we will calculate the sample size on the basis of a comparison of the measured primary outcome, the 6MWT result, between a small pilot study and a previous multimodal prehabilitation study conducted in our institute [15]. We will assume the average 6WMD in the aerobic prehabilitation group to be 30 m shorter than that in the multimodal group at 30 days postoperatively. The standard deviation (SD) for the 6MWD will be estimated to be 50 m for both groups. A sample size of 45 patients each in the aerobic group and multimodal prehabilitation group is required to detect a statistically significant difference at a two-sided significance level of 0.05 and statistical power of 90%. To account for patient dropout and missing data, we plan to recruit a total of 50 patients for each group.

### Data collection

Upon receiving informed consent, the researchers will collect the patients’ medical history and demographic information at the baseline interview. Baseline laboratory test results will then be retrieved from patients’ electronic medical records: complete blood count (CBC), liver and kidney function test results, electrolyte levels, and coagulation routine test results.

The 6MWD and pulmonary function test will be conducted and recorded by trained researchers at our institution after they provide standardized instructions and demonstrations of the tests. Patient-reported qualitative questionnaires will be administered by and the results will be recorded by trained researchers.

During the prehabilitation period, message interviews will be sent to patients twice a week by our researchers, and feedback on weekly prehabilitation performance will be recorded.

After surgery, designated clinicians will retrieve the surgical approach performed, tumor assessment results, pathology results, and postoperative complications from the patients’ electronic records.

All the data will be collected by researchers who have signed a confidential disclosure agreement. Patients’ identity and personal information will not be disclosed without consent.

All data collected will be recorded first in the paper-based case report form (CRF) and then entered into a digital database by an independent investigator. The digital database will be under the supervision of the Data Monitoring Committee (DMC) composed of two experienced doctors from the Department of Anesthesiology and one statistician from the Department of Medical Research Center, PUMCH, who are independent from this study.

### Oversight and monitoring

The Project Manage Group consists of two main researchers who are experienced anesthesiologist researchers. They will meet with other team members every 2 weeks to oversee work in various phases, discussing problems met during recruitment and follow-up. All research investigators comprise the Trial Steering Committee, and they will meet every half year to supervise the research progress and discuss if there is a need to make amendments to the protocol. The Ethics Committee and sponsor hold auditing every year to review conduct.

The DMC members will monitor the integrity of the data and patient safety. Upon data entry, the database will be locked by a password. DMC members will make the decision to unblind, conduct an interim analysis, or terminate the trial, if they deem it necessary. The DMC members meet every month to discuss exercise injuries and other safety risks.

### Statistical analysis

Patients’ baseline characteristics will be described by descriptive analysis and compared between the groups to detect clinically relevant differences. If evident baseline imbalance exists, the analysis of the primary outcome will be adjusted. For tests conducted perioperatively including 6MWD, pulmonary function test results, HADS and WHODAS 2.0 score, a linear mixed-effects model will be built to analyze the interaction between intervention and time [18]. Other secondary outcomes including the QoR-15 score, length of hospital stay, chest tube duration, and postoperative complications will be analyzed using *χ*^2^ test and the Mann–Whitney *U* where appropriate. Patients lost to follow-up will be considered as non-adherence. We will perform sensitivity analysis to test if the analyses of the primary outcome will be robust to the missing values. All statistical analyses will be completed in R (R Foundation for Statistical Computing, Vienna, Austria; version 3.5.2).

## Dissemination plans

The final trial dataset will be available on the online platform of the Chinese Academy of Medical Sciences, the sponsor. As this trial is unicentral, the main researchers will also have full access to the final trial dataset. Any data required to support the protocol can be supplied on request. We plan to communicate trial results thoroughly with our multidisciplinary team and encourage conversations with medical providers in other state facilities during the annual conference of the Chinese Medical Association. These medical providers will in turn disseminate results to the patient community. We will also inform the patient community of the results during pre-surgery evaluation and consults. We are planning to invite researchers from our multidisciplinary team as co-authors in the publication process, and we are not planning to use professional writers. For further dissemination of the results, we will post the study concept and major results on the website of the anesthesiology department of PUMCH. We will be open to provide the whole study protocol as well as the patient-level dataset when approached by interested health providers or researchers.

## Trial status

This manuscript is based on version 2.1 of our study protocol, updated on July 22, 2020. Enrollment started on November 9, 2019, then went on pause during January 2020 due to the COVID-19 pandemic. Since then, there were three spells of pandemic outbursts resulting in tightened restrictions on traveling and hospital admission policies, and recruitment was put on halt during those periods. Completion of recruitment is expected to be in November 2023. If there will be important modifications to protocols related to enrolment, prehabilitation strategies, outcomes, and statistics plans, we will inform all researchers and DMC members, report to our Institutional Review Board, and make timely updates on ClinicalTrials.gov and related journals.

## Discussion

The Prehabilitation in Thoracic Surgery Study investigates two types of prehabilitation strategies in patients undergoing VATS, a potentially more promising multimodal strategy and a more recognized aerobic exercise strategy, to answer the question of whether the former is more beneficial than the latter.

As gastrointestinal cancer is the most extensively explored condition regarding prehabilitation, there is a large amount of evidence showing that a multimodal strategy is more beneficial than a unimodal strategy in improving functional capacity recovery postsurgery. Our group formerly conducted a trial demonstrating that a short-term home-based multimodal prehabilitation strategy improves postsurgical 6MWD, a well-established marker for functional capacity. Most of the studies on prehabilitation in VATS patients included unimodal strategies, and aerobic exercise is widely considered to be the most important factor. We hope this study will shed more light on the differences in prehabilitation strategies in VATS patients.

This study is a pragmatic trial, and we made an effort to create a feasible and flexible protocol tailored to patients’ individual needs and preferences to promote adherence to training strategies and achieve viable, effective prehabilitation outcomes that will adapt to real-world clinical practice. The eligibility requirement of this trial consists of a wide range of patients’ characteristics, excluding mainly patients who are deemed unable or unsafe to perform the 6-min walk test and the prehabilitation program unsupervised due to serious comorbid conditions. The types of aerobic exercise, the elasticities of the pulling straps used in resistance training, and components in the psychological therapy are chosen by patients to facilitate adherence and ensure safety. Patient interviews make up a crucial part of the study, as they ensure patient safety and the fidelity of protocol adherence data and allow valuable feedback to be collected.

The training protocols for both arms were refined based on feedback from patients and multidisciplinary investigators during our previous study, so the strategy is more patient-centered and efficient. We propose a mixed methods evaluation panel to further assess the efficacy and feasibility of a multimodal prehabilitation strategy compared to aerobic training alone within a thoracoscopic surgery setting. We hope this study will provide more evidence on the prehabilitation methods within the thoracic surgery community and help promote better and faster functional recovery.

## Supplementary Information

**Additional file 1.** SPIRIT Checklist.

## Data Availability

Not applicable.

## Abbreviations

VATS: Video-assisted thoracoscopic surgery
PUMCH: Peking Union Medical College Hospital
6MWD: 6-Min walk distance
NSCLC: Non-small cell lung cancer
PPCs: Postoperative pulmonary complications
ASA: American Society of Anesthesiologists
6MWT: 6-Min walk test
FEV1: Forced expiratory volume in 1 s
FVC: Forced vital capacity
PEF: Peak expiratory flow
ALT: Alanine transaminase
CR: Creatine
CRP: C-reactive protein
Alb: Albumin
Glu: Glucose
HADS: Hospital Anxiety and Depression Scale
WHODAS 2.0: World Health Organization Disability Assessment Schedule II
QoR-15: Postoperative Quality of Recovery Score-15
RPE: Rating of perceived exertion
HR: Heart rate
ATS: American Thoracic Society
SD: Standard deviation
CBC: Complete blood count
CRF: Case report form
DMC: Data Monitoring Committee
