# Supplementary material for: The protocol for the prehabilitation for thoracic surgery study: a randomized pragmatic trial comparing a short home-based multimodal program to aerobic training in patients undergoing video-assisted thoracoscopic surgery lobectomy
Source: Trials. 2023 Mar 15;24:194. doi: 10.1186/s13063-023-07220-4 (PMC10017060; doi:10.1186/s13063-023-07220-4)
Supplement: Supplementary file 1 — Additional file 1. SPIRIT Checklist. [file 13063_2023_7220_MOESM1_ESM.doc]

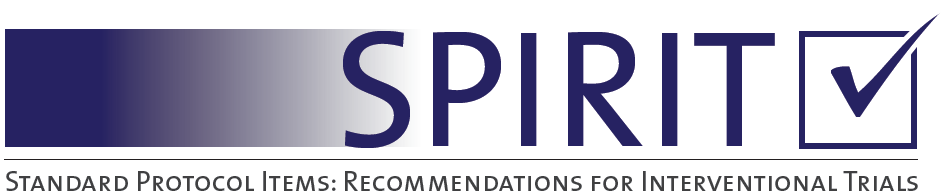


SPIRIT 2013 Checklist: Recommended items to address in a clinical trial protocol and related documents*

| Section/item | ItemNo | Description |
| --- | --- | --- |
| **Administrative information** | | |
| Title (page 1) | 1 | Line 1-3 |
| Trial registration  (Page 4) | 2 | Line 55-56 |
| Protocol version  (Page 19) | 3 | Line 374 |
| Funding  (Page 23) | 4 | Line 446-447 |
| Roles and responsibilities  (Pages 23, 18) | 5a | Line 452-455 |
|  | 5b | Line 446-447 |
|  | 5c | Line 447-449 |
|  | 5d | Line 333-343 (see Item 21a for data monitoring committee) |
| Introduction |  |  |
| Background and rationale  (Pages 3-6) | 6a | Line 62-73 |
|  | 6b | Line 74-94 |
| Objectives  (Page 6) | 7 | Line 97-100 |
| Trial design  (Pages 6-8) | 8 | Line 104-122  Explanation on “pragmatic trial design” at Line 398-408. |
| Methods: Participants, interventions, and outcomes | | |
| Study setting  (Page 7) | 9 | Line 111 |
| Eligibility criteria  (Pages 7,8,13) | 10 | Line 125-132  Line 111-113  Line 142-144  Line 239-241 |
| Interventions  (Pages 9-14) | 11a | Line 164-264 |
|  | 11b | Line 177-184 |
|  | 11c | Line 159-161; Line 169-176 |
|  | 11d | Line 164-167 |
| Outcomes  (Pages 14-15) | 12 | Line 266-289 |
| Participant timeline  (Page 16) | 13 | Line 291-296. |
| Sample size  (Page 16) | 14 | Line 298-307 |
| Recruitment  (Page 8) | 15 | Line 135-138 |
| **Methods: Assignment of interventions (for controlled trials)** | | |
| Allocation:  (Page 8) |  |  |
| Sequence generation | 16a | Line 138-141 |
| Allocation concealment mechanism | 16b | Line 141-142 |
| Implementation | 16c | Line 142-144 |
| Blinding (masking)  (Pages 7,18) | 17a | Line 119-122 |
|  | 17b | Line 122  Line 341-342 |
| **Methods: Data collection, management, and analysis** | | |
| Data collection methods  (Page 15) | 18a | Line 268-289 |
| (Pages 9,10,14) | 18b | Line 159-161  Line 169-172  Line 177-178  Line 255-257 |
| Data management  (Page 17) | 19 | Line 310-327 |
| Statistical methods  (Page 18) | 20a | Line 346-356 |
|  | 20b | Line 347-348  Line 353-355 |
| (Page 18) | 20c | Line 353-355 |
| **Methods: Monitoring** | | |
| Data monitoring  (Page 17) | 21a | Line 327-330  Line 340-341 |
| (Page 18) | 21b | There are no planned interim analyses. Line 341-342 |
| Harms  (Pages 10,18) | 22 | Line 177-178  Line 342-343 |
| Auditing  (Pages 17,18) | 23 | Line 333-343 |
| Ethics and dissemination | | |
| Research ethics approval  (Page 22) | 24 | Line 432-434 |
| Protocol amendments  (Page 20) | 25 | Line 378-382 |
| Consent or assent  (Page 8) | 26a | Line 137-138  Line 143-144 |
|  | 26b | This trial does not involve collecting biological specimens for storage. |
| Confidentiality  (Page 17) | 27 | Line 324-325 |
| Declaration of interests  (Page 23) | 28 | Line 443 |
| Access to data  (Page 19) | 29 | Line 359-362 |
| Ancillary and post-trial care  (Page 10) | 30 | Line 178-183 |
| Dissemination policy  (Page 19) | 31a | Line 362-369 |
| (Page 23) | 31b | Line 452-455 |
| (Page 19) | 31c | Line 369-371 |
| Appendices |  |  |
| Informed consent materials | 32 | The consent form will be uploaded separately. |
| Biological specimens | 33 | See above 26b there will be no biological specimens collected |

*It is strongly recommended that this checklist be read in conjunction with the SPIRIT 2013 Explanation & Elaboration for important clarification on the items. Amendments to the protocol should be tracked and dated. The SPIRIT checklist is copyrighted by the SPIRIT Group under the Creative Commons “[Attribution-NonCommercial-NoDerivs 3.0 Unported](http://www.creativecommons.org/licenses/by-nc-nd/3.0/)” license.
